# Tumor antigens across cancer progression: from immune recognition and selection to precision cancer immunotherapy

**DOI:** 10.3389/fimmu.2026.1939919

**Published:** 2026-10-12

**Authors:** Stanley P. Leong

**Affiliations:** 1 Department of Surgery and Melanoma Center, California Pacific Medical Center, San Francisco, CA, United States; 2 Department of Surgery, University of California, San Francisco, San Francisco, CA, United States

**Keywords:** antigen presentation, cancer immunoediting, immune escape, immunopeptidomics, precision cancer therapy, spatial biology, tumor antigens, tumor microenvironment

## Abstract

Tumor antigens provide the molecular interface between malignant transformation and host immunity, but their biological significance is determined by more than their molecular existence. Antigen clonality, abundance, processing, human leukocyte antigen (HLA) presentation, dendritic-cell function, T-cell recognition, spatial immune organization, and the surrounding tumor microenvironment (TME) collectively determine whether an antigen becomes immunologically visible and therapeutically actionable. Across cancer progression, tumor-cell antigenic states and the TME undergo reciprocal selection: immune pressure can eliminate highly visible clones while favoring antigen-loss variants, HLA defects, immune exclusion, suppressive myeloid and stromal states, and treatment-resistant populations. Recent single-cell, spatial and three-dimensional imaging, as well as immunopeptidomic studies show that this evolution is heterogeneous across tumor regions and anatomical compartments rather than a uniform linear process. This review therefore advances an antigen-TME co-evolution framework linking primary tumors, lymphatic and nodal immune compartments, systemic dissemination, distant metastatic niches, therapy, minimal residual disease, and recurrence. It critically evaluates the strengths and limitations of the classical immunoediting model; compares melanoma, breast, colorectal, lung, prostate, sarcoma, and virus-associated cancers according to antigen source, clonality, presentation competence, immune architecture, and dominant therapeutic bottleneck; and examines clinical translation through checkpoint blockade, cellular therapy, vaccines, antibodies, antibody-drug conjugates, immunopeptidomics, and computational antigen prediction. Particular attention is given to the gap between predicted peptide binding and verified presentation or immunogenicity, the need for longitudinal rather than single-biopsy profiling, and the requirement for prospective validation of artificial-intelligence models. The central conclusion is that tumor antigens become clinically meaningful only when antigenic state and microenvironmental context are assessed together, providing a more rigorous foundation for precision cancer immunotherapy.

## 1 Introduction

Tumor antigens represent the central biological interface between malignant transformation and host immunity. As normal cells acquire oncogenic alterations, they generate or aberrantly express molecular structures that distinguish them from their nonmalignant counterparts, including mutation-derived, lineage-associated, cancer-testis, viral, fusion-derived, and noncanonical antigens (1–4). Once processed and presented by human leukocyte antigen (HLA) molecules, tumor-derived peptides may be recognized by T lymphocytes and initiate adaptive antitumor immune responses (4, 5). In this way, tumor antigens provide the molecular foundation for immune surveillance, immune editing, immune-checkpoint blockade, cancer vaccines, adoptive cellular therapy, and other antigen-directed forms of precision cancer therapy (6–10).

The immunologic significance of tumor antigens is determined not only by their origin, clonality, abundance, processing, HLA binding, and T-cell receptor recognition, but also by the tumor microenvironment (TME) in which antigen recognition occurs. Dendritic-cell function, adaptive and innate immunity, stromal and vascular architecture, metabolic state, and immune-checkpoint regulation collectively influence whether tumor antigens generate immune activation, suppression, or escape (11–18). Innate immune cells generally do not recognize tumor antigens through the conventional peptide-HLA-restricted mechanisms characteristic of alpha-beta T cells, although unconventional lymphocyte populations, including gamma-delta T cells and MR1-restricted mucosal-associated invariant T cells, provide important exceptions to a simple innate-versus-adaptive dichotomy. Innate sensing of cellular stress, missing-self signals, damage-associated molecular patterns, inflammatory mediators, and tumor-derived nucleic acids can promote tumor-cell elimination, antigen uptake, dendritic-cell maturation, type I interferon signaling, and T-cell priming, but these responses may also be redirected by the TME toward immune suppression and tumor progression (19–23).

The organizing contribution of this review is to map tumor-antigen evolution and TME-mediated selection across successive anatomical compartments of cancer progression and dissemination. Cancer immunoediting established the principle that immunity can both restrain malignancy and select immune-resistant variants, while the cancer-immunity cycle and cancer-immune set point define functional requirements for effective antitumor immunity (7, 8, 11, 18). More recent genomic, single-cell, spatial, and multilayer models emphasize that tumor and immune heterogeneity generate temporally and spatially distinct immunological niches (24–27). Building on these concepts, the present framework follows the antigen-bearing cancer cell through the primary tumor, tumor-lymphatic interface, sentinel and regional lymph nodes, circulation, distant metastatic sites, treatment-altered tissues, residual disease, and recurrence. The anatomical organization is based on a recently proposed two-route framework of lymphatic and hematogenous dissemination, with both pathways ultimately entering the venous circulation before cardiopulmonary transit and systemic arterial distribution (28); primary evolutionary studies further support biological distinctions among lymphatic, nodal, and distant metastatic compartments (29, 30).

This framework also generates testable predictions. First, the same antigenically defined tumor clone may have different immune fates in different anatomical compartments if local HLA presentation, immune access, stromal organization, or suppressive programs differ. Second, antigen sequence or abundance alone should incompletely predict immune escape when HLA presentation and spatial immune access are not measured concurrently. Third, antigen-TME profiling of sentinel or regional lymph nodes may provide biological information not captured by the primary tumor alone. Findings showing invariant immune behavior across compartments despite marked microenvironmental differences, no reproducible information added by nodal profiling, or complete prediction of immune response from sequence-level antigen measurements alone would argue against important components of this framework. Recent single-cell and spatial studies provide the experimental foundation for testing these propositions by resolving structured cellular neighborhoods and immune states within and across anatomical sites (31–35).

Tumor antigens and the TME evolve reciprocally during cancer progression. Through cancer immunoediting, highly immunogenic tumor-cell populations may be eliminated or restrained, whereas variants with reduced antigenicity, impaired antigen presentation, checkpoint-mediated suppression, resistance to cytotoxicity, or protection within suppressive microenvironmental niches may acquire a selective advantage (7, 8, 24, 36, 37). These processes generate antigen loss, HLA defects, impaired interferon signaling, T-cell exhaustion, suppressive myeloid states, stromal barriers, metabolic constraints, and spatially heterogeneous immune escape (4, 25, 38–40). Therapeutically, tumor antigens may serve as direct targets of immune-checkpoint blockade, adoptive cellular therapy, tumor-infiltrating lymphocyte therapy, engineered T-cell therapies, cancer vaccines, monoclonal antibodies, and antibody-drug conjugates, while chemotherapy, radiation therapy, targeted therapy, endocrine therapy, and rational combinations may alter antigen release, antigen presentation, immune recognition, and the TME (41–47).

This review examines tumor antigens across cancer progression as dynamic determinants of immune recognition, immune selection, immune escape, therapeutic response, and precision cancer therapy. Section 2 reviews tumor-antigen origin, HLA-dependent processing and presentation, immunopeptidomics, antigen-presentation defects, and antigenic heterogeneity. Sections 3 and 4 examine innate and adaptive antitumor immunity across tumors and lymphoid compartments. Section 5 critically evaluates tumor-antigen evolution, immunoediting, spatial selection, antigen loss, and resistance. Section 6 addresses therapeutic targeting and the clinical translation of antigen discovery and prediction. Section 7 compares major human cancers analytically according to antigen source, presentation competence, immune architecture, and dominant therapeutic bottleneck. Section 8 defines actionable field priorities involving longitudinal profiling, spatial biology, immunopeptidomics, artificial intelligence, functional validation, and mechanism-based patient selection.

The present review offers an integrated, compartment-by-compartment framework linking tumor-antigen evolution, immune selection, the tumor microenvironment, anatomical dissemination, treatment pressure, residual disease, recurrence, and therapeutic resistance across the metastatic cascade. Its distinctive contribution lies in bringing these processes together as a continuous biological and clinical continuum rather than treating them as isolated events within separate anatomical compartments or therapeutic contexts. This framework therefore provides an organizing structure for examining how antigenic state and microenvironmental context co-evolve from early transformation through regional and systemic dissemination, treatment-associated selection, residual disease, and recurrence across the metastatic cascade.

### 1.1 Literature search and review approach

This narrative review was developed through searches of PubMed/MEDLINE and complementary review of reference lists from relevant primary studies and major reviews. Literature was considered through August 2026, with emphasis on tumor antigens and neoantigens, HLA-dependent antigen presentation, immunopeptidomics, cancer immunoediting, the tumor microenvironment, single-cell and spatial biology, lymphatic and nodal immunity, metastatic evolution, immune-checkpoint therapy, cellular therapy, cancer vaccines, antibody-based therapy, and computational antigen prediction. Landmark mechanistic studies were retained where they established fundamental concepts, while recent primary, translational, and clinical studies were prioritized for evolving technologies and therapeutic applications. Particular attention was given to studies linking antigenic state with immune architecture, anatomical compartment, treatment response, or immune escape. This was not a systematic review or meta-analysis, and no formal risk-of-bias instrument or predefined study-selection algorithm was applied. Accordingly, literature selection reflects a narrative synthesis and may be subject to selection bias despite efforts to include supportive, conflicting, and limiting evidence.

## 2 Tumor antigens and antigen presentation

Tumor antigens are dynamic biological entities that evolve during malignant transformation, tumor progression, metastatic dissemination, therapeutic intervention, dormancy, and recurrence. Their immunologic significance depends not only on molecular origin, but also on antigen processing, HLA-dependent presentation, adaptive immune recognition, and regulation by the surrounding TME (**Figure 1**). Across the metastatic cascade, these changes occur within sequential anatomical compartments reached through lymphatic and hematogenous dissemination (28). The cellular composition of the primary, lymphatic, nodal, circulating, metastatic, and therapy-associated microenvironments is summarized in **Supplementary Table S1**, whereas the corresponding tumor-antigen features, biomarkers, cytokines, chemokines, immune checkpoints, and signaling programs are summarized in **Supplementary Table S2**. Together, antigen generation, processing, presentation, and microenvironmental regulation determine whether malignant cells are recognized, eliminated, edited, tolerated, or permitted to escape immune control (3–5, 8, 11, 36).

**Figure 1:**
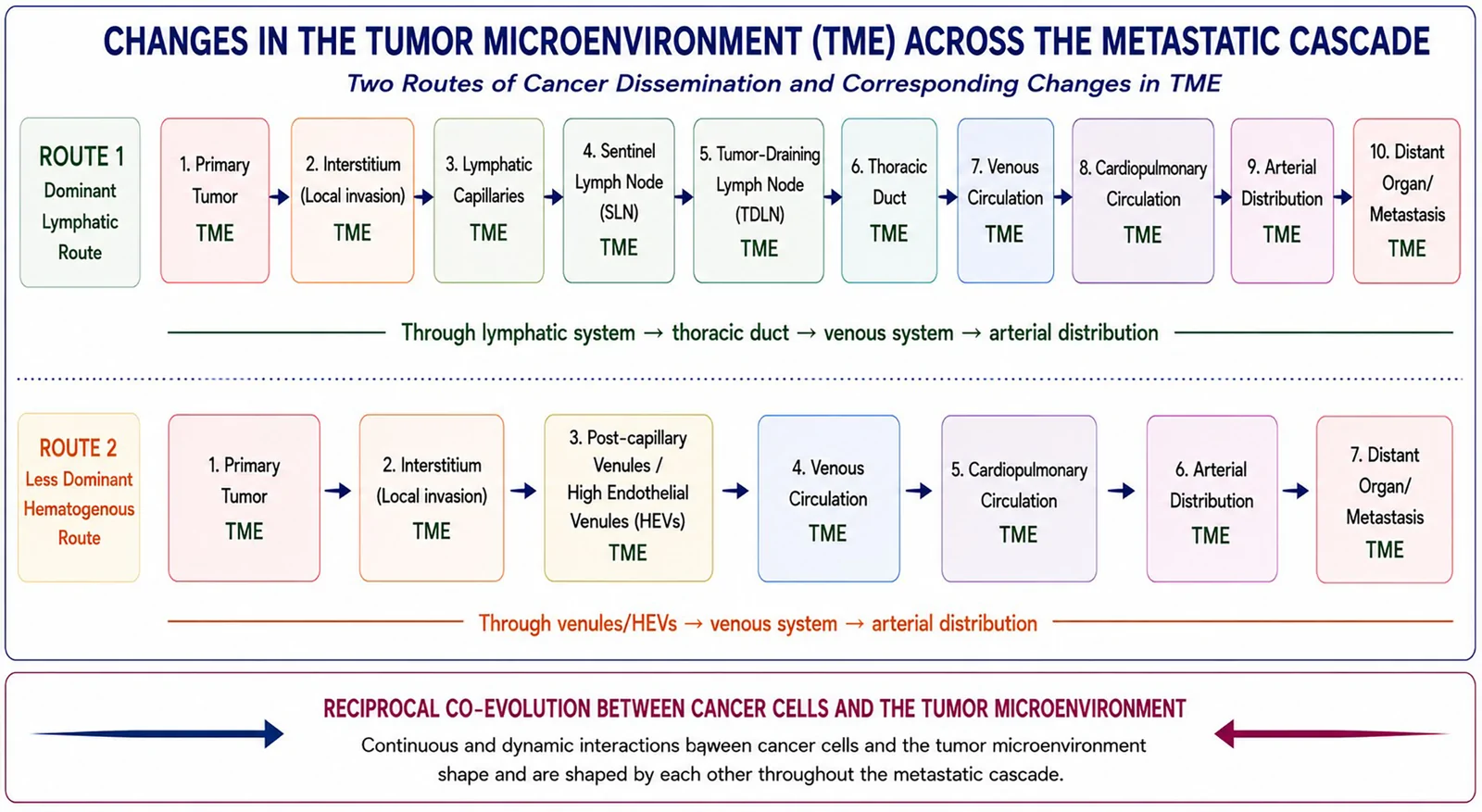
Changes in the tumor microenvironment across the metastatic cascade. The figure illustrates two anatomical routes of cancer dissemination and the reciprocal co-evolution of cancer cells and the tumor microenvironment (TME) across disease progression. Route 1 depicts lymphatic dissemination from the primary tumor through the tumor-lymphatic interface, sentinel and regional lymph nodes, followed by lymphatic return to the venous circulation, cardiopulmonary transit, and systemic arterial distribution to distant organs. Route 2 depicts direct hematogenous dissemination through venous entry, cardiopulmonary transit, and subsequent systemic arterial distribution. The anatomical depiction follows a recently proposed two-route framework of cancer dissemination (28), while primary evolutionary studies further support biological distinctions among lymphatic, nodal, and distant metastatic compartments (29, 30). Across both routes, cancer cells undergo continuous antigenic and phenotypic evolution while interacting with immune, stromal, vascular, and lymphatic components of the TME (**Figure 2**). The third row emphasizes the central concept of reciprocal cancer-cell-TME co-evolution throughout the metastatic cascade.

Tumor antigens arise through diverse mechanisms, including somatic mutation, chromosomal instability, gene fusion, aberrant gene expression, epigenetic dysregulation, oncogenic viral infection, post-translational modification, alternative splicing, defective translation, and re-expression of developmental or lineage-associated proteins. These processes generate neoantigens, tumor-associated antigens, cancer-testis antigens, differentiation antigens, viral antigens, fusion proteins, aberrantly glycosylated proteins, and other modified cellular proteins (1–4, 24, 49, 50). Neoantigens derived from tumor-specific mutations are attractive because they are absent from the normal germline proteome, although their therapeutic value depends on whether the mutant peptide is actually expressed, processed, presented, recognized, and retained across tumor cells.

The distinction between a predicted antigen and a presented immunogenic antigen is fundamental. DNA or RNA sequencing can nominate thousands of mutant peptide candidates, but only a minority are processed into HLA-bound peptides and an even smaller fraction elicit measurable T-cell responses. Mass-spectrometry-based immunopeptidomics directly samples HLA-presented peptides and can therefore verify a biological step that sequence-based prediction only infers. Integrated sequencing and mass spectrometry identified immunogenic mutant peptides in experimental models and subsequently demonstrated directly presented neoepitopes in native human melanoma tissue (51, 52). Large HLA peptidome datasets and improved prediction algorithms have increased coverage across HLA alleles, but sensitivity remains constrained by tissue input, peptide abundance, instrument performance, HLA expression, and sampling heterogeneity (53–55).

Recent advances in next-generation sequencing, single-cell analysis, spatial biology, multi-omics profiling, and immunopeptidomics have shown that antigenic heterogeneity is a fundamental characteristic of human cancer. Individual tumors often contain multiple subclonal populations with distinct antigenic repertoires, and metastatic lesions may differ from primary tumors because of genetic diversification, immune editing, tissue-specific adaptation, and therapy-induced selection (24, 27, 29, 56). Spatial studies further identify cellular neighborhoods (recurrent local arrangements of interacting cell types), immune hubs (spatially organized multicellular structures enriched for coordinated immune interactions), and ecotypes (recurrent combinations of malignant and microenvironmental cell states) that vary among patients and tumor regions (31–35). These observations move antigen evolution beyond a purely genomic model: the biological consequence of an antigen depends on where antigen-bearing cells are located and which immune and stromal populations can interact with them.

The presence of tumor antigens alone is insufficient to generate effective antitumor immunity. In the HLA class I pathway, intracellular proteins are degraded by the proteasome, peptides are transported into the endoplasmic reticulum through transporter-associated antigen-processing proteins, loaded onto HLA class I molecules, and transported to the cell surface for recognition by CD8+ cytotoxic T lymphocytes (5, 57, 58). Because virtually all nucleated cells express HLA class I molecules, this pathway enables continuous immune surveillance of malignant cells by cytotoxic T cells.

In contrast, extracellular antigens are internalized by professional antigen-presenting cells, particularly dendritic cells, macrophages, and B lymphocytes, which constitutively express HLA class II molecules. Within endosomal and lysosomal compartments, these proteins are degraded into peptide fragments, loaded onto HLA class II molecules, and transported to the cell surface for recognition by CD4+ T lymphocytes (5, 57, 58). After antigen recognition, costimulation, and cytokine exposure, CD4+ T cells may differentiate into Th1, Th2, Th17, T follicular helper, or regulatory T-cell subsets. These subsets support CD8+ T-cell expansion, B-cell maturation, macrophage and dendritic-cell regulation, inflammatory control, or immune suppression, depending on the antigenic signal and surrounding TME (12, 57, 58).

Dendritic cells add a crucial cross-presentation pathway: exogenous tumor-derived antigens can be presented on HLA class I molecules to naive CD8+ T cells. Effective priming therefore requires more than peptide-HLA binding; it also requires dendritic-cell maturation, costimulatory signaling, migration to appropriate lymphoid compartments, and cytokine support (11, 22, 23). This is especially relevant at the tumor-lymphatic interface and within tumor-draining or sentinel lymph nodes, where antigen transport, dendritic cells, naive lymphocytes, high endothelial venules, lymphatic sinuses, and nodal stroma converge.

The effectiveness of antigen presentation is further shaped by HLA diversity. HLA polymorphisms determine which antigenic peptides can be efficiently presented and therefore influence the breadth and quality of antitumor immune recognition. HLA genotype and the capacity to present diverse neoantigen repertoires have been associated with outcomes after immune-checkpoint blockade (45, 59). However, HLA genotype is a permissive constraint rather than a complete biomarker because peptide generation, HLA expression, clonality, T-cell repertoire, and TME state remain necessary downstream determinants.

Tumors frequently impair HLA-dependent antigen presentation through loss or downregulation of HLA class I, beta2-microglobulin mutations, altered proteasomal processing, defects in transporter-associated antigen processing, impaired interferon signaling, or suppression of antigen-presenting-cell function (4, 38, 39, 60). The TME can further suppress presentation through inhibitory cytokines, regulatory immune populations, myeloid dysfunction, metabolic stress, hypoxia, and checkpoint signaling (13, 25, 61). Thus, a tumor may contain potentially immunogenic antigens while remaining functionally invisible to T cells.

Importantly, antigen presentation is itself subject to evolutionary pressure. Tumor-cell populations that retain highly immunogenic antigens and efficient HLA presentation may be preferentially eliminated, whereas variants that reduce antigen expression, lose antigenic targets, impair HLA presentation, resist interferon-mediated immune signaling, or recruit suppressive microenvironmental elements may gain a selective advantage (4, 8, 24, 26, 36, 62). The resulting selection can differ among regions of the same tumor and among primary, nodal, and distant sites, making longitudinal and multisite assessment particularly important.

## 3 Innate immunity and antitumor responses

Innate immunity provides the earliest host response to malignant transformation and conditions subsequent adaptive antitumor immunity. Unlike conventional alpha-beta T-cell responses, innate sensing is not generally dependent on antigen-specific peptide-HLA recognition; instead, it detects cellular stress, tissue injury, altered-self or missing-self signals, damage-associated molecular patterns, abnormal nucleic acids, cytokines, and chemokines (11, 19, 57, 58). These responses can promote tumor-cell elimination, antigen uptake, dendritic-cell maturation, and inflammatory priming, but the TME can redirect them toward immune suppression and tumor progression (11, 22, 23).

NK cells, macrophages, neutrophils, dendritic cells, innate lymphoid cells, mast cells, and other myeloid populations have context-dependent roles in cancer. Their functional states are shaped by local cytokines, metabolites, stromal signals, vascular and lymphatic changes, and therapy-induced injury; therefore, abundance alone does not establish whether an innate population is tumor-restraining or tumor-promoting (13, 25, 61, 63, 64).

### 3.1 Natural killer cells

Natural killer cells recognize transformed cells through the balance of activating and inhibitory receptors that monitor HLA class I expression and stress-induced ligands (19, 57, 58, 64). Loss of HLA class I may reduce CD8+ T-cell recognition while increasing susceptibility to NK-cell missing-self recognition. NK-cell cytotoxicity and interferon-gamma production can support antitumor immunity, but TGF-beta, adenosine, hypoxia, suppressive myeloid cells, and regulatory populations can inhibit these functions (13, 25, 61).

### 3.2 Macrophages and neutrophils

Macrophages and neutrophils illustrate the plasticity of innate immunity within tumors. Macrophages can support tumor-cell killing and antigen presentation or promote immune suppression, angiogenesis, lymphangiogenesis, matrix remodeling, invasion, and resistance; the traditional M1/M2 terminology therefore incompletely represents the continuum observed in human tumors (13, 25, 63, 65). Neutrophils can likewise exert inflammatory or cytotoxic effects while also supporting matrix remodeling, immunosuppression, circulating tumor-cell survival, and metastasis (13, 25, 66).

### 3.3 Additional innate immune populations

Additional innate lymphoid, mast-cell, and myeloid populations contribute to cytokine production, vascular regulation, matrix remodeling, and recruitment of other immune cells. Their relevance to tumor-antigen biology is principally through the microenvironmental conditions they create for antigen release, presentation, immune access, or suppression (13, 25, 63, 64).

### 3.4 Innate immune sensing of tumors

Innate immune responses to cancer are initiated through recognition of cellular stress, tissue injury, abnormal nucleic acids, and damage-associated molecular patterns released by stressed or dying tumor cells. Pattern-recognition pathways include Toll-like, NOD-like, RIG-I-like, and cGAS-STING pathways (11, 20, 21, 57). Activation promotes type I interferon production, inflammatory cytokines, chemokines, dendritic-cell maturation, and antigen presentation. Radiation therapy, chemotherapy, and selected targeted therapies can increase tumor-cell stress and antigen release, although the magnitude and durability of productive immune activation vary among tumors (41–43).

### 3.5 Dendritic cells: bridging innate and adaptive immunity

Dendritic cells occupy a uniquely important position because they link innate tumor sensing with adaptive antigen-specific immunity. They survey the TME for damage-associated signals and tumor-derived material, then mature, process antigens, express costimulatory molecules, and migrate through lymphatics to regional lymphoid organs (11, 22, 23). Conventional type 1 dendritic cells are particularly important for cross-presentation to CD8+ T cells. In sentinel and tumor-draining lymph nodes, dendritic-cell function can therefore determine whether tumor-antigen drainage leads to productive priming or to tolerance and immune suppression (22, 23, 48).

**Figure 1** places these immune and stromal interactions within the broader anatomical framework of cancer dissemination. By linking the two routes of metastatic spread with reciprocal co-evolution between cancer cells and the TME, the figure emphasizes that antigen information and immune selection evolve within a multicellular network rather than through a single tumor-cell-only pathway.

## 4 Adaptive immunity and antitumor responses

Adaptive immunity supplies the antigen-specific arm of antitumor defense through HLA-restricted recognition, clonal expansion, effector function, and immunologic memory. CD8+ and CD4+ T cells, B cells, plasma cells, memory populations, antigen-presenting cells, and checkpoint pathways interact within tumors, draining lymph nodes, tertiary lymphoid structures (TLSs), and metastatic niches (8, 11, 13–17, 36, 57, 58). Thus, the anatomical compartment in which an antigen is encountered can be as important as the antigen itself in determining activation, tolerance, exhaustion, exclusion, memory, or escape.

This compartmental organization is particularly important because antigen-specific responses can be initiated, amplified, suppressed, or redirected as tumor material moves from the primary site through lymphatic and nodal compartments and into distant metastatic environments. Adaptive immunity therefore functions not only as an effector system but also as an evolutionary pressure on antigen-bearing tumor populations.

CD8+ cytotoxic T lymphocytes recognize tumor-derived peptides on HLA class I molecules and eliminate susceptible cells through cytotoxic and cytokine-dependent mechanisms (5, 11, 57, 58). Their selective pressure is biologically important: tumors enriched for clonal neoantigens may support more coherent antitumor responses, whereas subclonal, poorly presented, or lost neoantigens can permit escape (26, 62).

CD4+ T lymphocytes recognize peptides presented on HLA class II molecules and differentiate according to antigenic signal, costimulation, cytokine exposure, and TME context (12, 57, 58). Helper programs can support CD8+ T-cell activity, B-cell maturation, germinal-center reactions, and macrophage or dendritic-cell function, whereas regulatory T cells can suppress effector immunity through cytokines, metabolic competition, checkpoint pathways, and direct inhibition.

B lymphocytes can produce tumor-reactive antibodies, present antigen, regulate T-cell activation, and contribute to TLS organization. Mature B-cell-rich TLSs have been associated with favorable outcome and checkpoint-blockade response in several cancers (14–17), but their functional significance depends on maturation, spatial relationship to tumor, high endothelial venules, dendritic-cell content, and local immune state rather than on B- or T-cell counts alone.

Long-lived memory T and B cells can mediate rapid responses after subsequent antigen exposure (67). In cancer, durable memory may contribute to prolonged control after checkpoint blockade, adoptive cellular therapy, or vaccination, whereas failure to maintain antigen-specific memory may permit residual disease or recurrence. Memory therefore represents another biological filter between initial antigen recognition and durable clinical benefit.

## 5 Tumor-antigen evolution, immunoediting, and immune escape

The development of clinically apparent cancer reflects not only acquisition of malignant characteristics by tumor cells, but also progressive selection under host immunity and the TME. Cancer immunoediting links tumor-antigen evolution, immune selection, and cancer progression and is conventionally described as elimination, equilibrium, and escape (7, 8, 36, 37). During elimination, immunogenic cells may be destroyed; during equilibrium, immune pressure restrains outgrowth while selecting resistant variants; during escape, populations with reduced antigenicity, impaired presentation, resistance to cytotoxicity, or microenvironmental protection can progress.

A central mechanistic refinement is that immunoediting does not act on antigen sequence alone. Selection can occur at multiple layers: mutation and clonality, transcription, translation, proteasomal processing, HLA binding, HLA abundance, interferon responsiveness, spatial access to immune effectors, and susceptibility to cytotoxicity. Multiregion studies in lung cancer provide evidence consistent with immune pressure shaping neoantigen landscapes and selecting escape mechanisms, including loss of neoantigens and alterations in antigen presentation (26, 62). The resulting tumor may therefore retain a high nominal mutational burden while becoming functionally less visible to T cells.

Single-cell and spatial technologies add another dimension. Multiplexed imaging in triple-negative breast cancer identified immune-mixed and immune-compartmentalized tumors with spatially patterned checkpoint expression; CODEX analysis in colorectal cancer identified coordinated cellular neighborhoods at the invasive front; single-cell and spatial transcriptomics revealed multicellular immune hubs and breast-cancer ecotypes; and three-dimensional imaging demonstrated continuous spatial gradients rather than sharply bounded immune states (31–35). These studies show that immunoediting may be spatially local: a clone can be immunologically exposed in one neighborhood yet protected in another.

Tumors also escape by disrupting HLA-dependent presentation. Loss or downregulation of HLA class I, beta2-microglobulin mutation, altered proteasomal processing, transporter defects, and impaired interferon signaling can reduce T-cell recognition despite continued antigen expression (4, 38, 39, 60). Sustained immune and therapeutic pressure can further favor antigen-negative or antigen-low variants. In this setting, apparent ‘antigen loss’ may reflect true genomic deletion, transcriptional silencing, altered processing, reduced HLA display, or spatial sampling of a heterogeneous tumor; these mechanisms are biologically distinct and require different therapeutic responses.

T-cell exhaustion is another consequence of chronic antigen exposure in a suppressive TME. Persistent stimulation together with checkpoint signaling, inadequate costimulation, metabolic stress, and suppressive cytokines produces altered transcriptional and epigenetic states with reduced proliferation and effector function (40, 41, 68, 69). Checkpoint blockade can reinvigorate selected T-cell states but cannot restore recognition when the relevant antigen-HLA complex is absent or when T cells cannot access tumor cells.

Regulatory T cells, myeloid-derived suppressor cells, tumor-associated macrophages, cancer-associated fibroblasts, endothelial cells, extracellular matrix, hypoxia, adenosine, and nutrient competition can impair antigen presentation, lymphocyte trafficking, and effector function (13, 25, 61, 70). Accordingly, immune escape may be antigen-intrinsic, presentation-dependent, spatial, or microenvironmental, and multiple mechanisms can coexist within the same patient.

### 5.1 Critical appraisal of the immunoediting model and antigen-centric explanations

The elimination-equilibrium-escape model remains strongly supported as an organizing framework because it correctly predicts that host immunity can eliminate immunogenic tumor cells, maintain residual populations under selective pressure, and favor emergence of resistant variants (7, 8, 36, 37). Experimental models provide the clearest causal evidence for these processes, whereas human genomic, multiregion, and longitudinal studies provide evidence consistent with immune-mediated selection during tumor evolution. Its principal limitation is therefore not that immunoediting is incorrect, but that the classical three-phase formulation does not fully capture the spatial, anatomical, clonal, and temporal heterogeneity observed in human cancer. Elimination, equilibrium, and escape may occur simultaneously in different regions or clones, and metastatic or therapy-associated compartments may impose distinct selective pressures (27, 29, 30, 56).

A second limitation is the tendency to equate mutation burden with antigenicity. Tumor mutational burden is a population-level correlate in selected settings, but clonal distribution, expression, processing, HLA genotype, HLA loss, and T-cell recognition determine whether mutations generate effective immune targets (45, 50, 59, 62, 71). Conversely, low-mutational-burden cancers can contain actionable viral, lineage, fusion-derived, or noncanonical antigens. Antigen-centric models therefore require explicit integration of presentation and TME context rather than assuming that more mutations necessarily create more effective immunity.

A third controversy concerns whether immune escape should be interpreted primarily as cancer-cell evolution or as tissue-level immune dysfunction. The evidence supports both. Genomic and HLA alterations can make cancer cells intrinsically less visible, while spatial exclusion, suppressive myeloid states, abnormal vasculature, and metabolic competition can prevent otherwise competent antigen-specific T cells from functioning (4, 13, 31, 33, 61). The antigen-TME co-evolution model used here therefore treats immune escape as a coupled system rather than assigning primacy to one compartment.

**Table 1** summarizes how tumor-antigen characteristics, immune consequences, and clinical implications can vary across disease states and anatomical compartments. The rows are organizational rather than strictly temporal: elimination, equilibrium, escape, treatment selection, and microenvironmental protection may coexist across different regions and tumor subclones.

**Table 1:** Evolution of tumor antigens across disease states and anatomical compartments.

Disease state/ anatomical compartment	Tumor-antigen characteristics	Immune consequences	Clinical implications
Early transformation	Emergence of novel tumor antigens and neoantigens from genetic, epigenetic, viral, and oncogenic alterations (3, 6, 7)	Initiation of immune recognition and immunosurveillance	Potential elimination before clinical detection; early selection of poorly visible variants
Primary tumor growth	Increasing antigen diversity, clonal expansion, subclonal heterogeneity, and variable HLA presentation (8, 24, 25, 62)	Activation of innate/adaptive immunity with spatially heterogeneous immunoediting	Selection of less immunogenic, poorly presented, excluded, or immune-resistant populations
Local progression	Antigen modulation, HLA/APM defects, subclonal evolution, and spatially organized immune/stromal neighborhoods (4, 24, 31, 32, 56)	Simultaneous elimination, equilibrium, and escape in different tumor regions	Local invasion and treatment resistance despite retained antigenicity in selected regions
Lymphatic/nodal dissemination	Antigen drainage and presentation in tumor-draining nodes; nodal clonal diversification; potential tolerance or productive priming (29, 30, 48)	Regional immune selection and altered antigen presentation	Nodal metastasis can function as both immune interface and metastatic niche
Distant metastasis	Organ-adapted antigenic states and site-specific immune/stromal selection (29, 30, 72)	Altered recognition and organ-specific immune escape	Metastatic lesions may require site-specific biomarker and therapeutic assessment
Residual disease/dormancy	Persistence of antigen-low, poorly presented, treatment-tolerant, or protected tumor cells (24, 25, 73)	Immune equilibrium, partial control, or evasion	Risk of later recurrence; longitudinal profiling needed
Recurrence/resistance	Antigen-loss variants, HLA/APM defects, checkpoint activation, resistant clones, and treatment-modified TME (4, 26, 38, 39, 60)	Immune escape and diminished therapeutic responsiveness	Mechanism-specific reassessment rather than assuming the pretreatment target persists

The table summarizes antigenic and microenvironmental selection from transformation through recurrence. Rows are organizational rather than strictly temporal; immunoediting, equilibrium, escape, treatment selection, and microenvironmental protection may occur concurrently across different tumor regions, subclones, and anatomical compartments.

## 6 Antigens as therapeutic targets: foundations of precision cancer therapy

The recognition that tumor antigens can serve as therapeutic targets has transformed cancer treatment. Immunotherapeutic approaches directly exploit antigen-specific immune mechanisms, while antibodies and antibody-drug conjugates use surface antigen expression as a molecular address. Chemotherapy, radiation therapy, targeted therapy, and endocrine therapy can indirectly modify antigen release, presentation, and the TME (25, 41–44, 46).

### 6.1 Tumor antigens as direct targets of immunotherapy

Immune-checkpoint blockade has achieved the broadest clinical impact among antigen-dependent immunotherapies. CTLA-4 blockade enhances T-cell priming and expansion, whereas PD-1/PD-L1 blockade acts predominantly on effector responses in the TME (41, 68, 74). Checkpoint inhibitors do not directly target tumor antigens; efficacy still requires antigen availability, presentation, clonotypes capable of recognition, tumor access, and sufficient effector function. Additional inhibitory pathways including LAG-3, TIM-3, TIGIT, and VISTA participate in overlapping regulatory networks (40, 69, 75).

Adoptive cellular therapies provide more direct antigen targeting. Tumor-infiltrating lymphocyte therapy expands naturally occurring tumor-reactive cells; engineered T-cell receptor therapies target intracellular antigens presented by HLA; and chimeric antigen receptor T cells recognize surface antigens independently of HLA restriction (9, 76, 77). These distinctions are clinically important because loss of HLA presentation undermines TCR-based strategies but does not necessarily prevent CAR-based recognition, whereas heterogeneous or low surface-antigen density can limit CAR or antibody approaches.

Bispecific T-cell engagers provide another antigen-directed strategy by linking a tumor-associated surface antigen to CD3 on T cells and redirecting cytotoxicity without requiring conventional peptide-HLA recognition. Their activity illustrates the importance of target density, antigen heterogeneity, T-cell availability, immune suppression within the TME, and on-target/off-tumor safety; cytokine-release syndrome and antigen-loss escape remain clinically relevant constraints (78).

Cancer vaccines seek to increase immunity against tumor-associated antigens or patient-specific neoantigens. Early personalized vaccine studies established feasibility and immunogenicity (10, 79, 80). In resected high-risk melanoma, KEYNOTE-942 was an open-label randomized phase 2b study of 157 patients assigned 2:1 to individualized mRNA-4157 (V940) plus pembrolizumab or pembrolizumab alone; recurrence-free survival favored the combination (HR for recurrence or death, 0.561; 95% CI, 0.309–1.017), while overall-survival evidence remained immature (81). Personalized RNA vaccination in pancreatic cancer generated robust neoantigen-specific T-cell responses (82). In high-risk clear-cell renal cell carcinoma, a phase I study of nine patients demonstrated feasibility and strong patient-specific immune responses, with no recurrences at a median 40.2 months of follow-up; because the study was small and uncontrolled, these observations should be interpreted as early feasibility and immunogenicity evidence rather than definitive comparative efficacy (83).

### 6.2 Immunopeptidomics and the clinical translation of antigen discovery

Immunopeptidomics, the direct measurement of the repertoire of peptides presented by HLA molecules, addresses a central weakness of sequence-only neoantigen prediction. In native human melanoma, mass spectrometry identified presented mutant peptides recognized by tumor-reactive T cells (52), while lung-cancer studies demonstrated regionally heterogeneous presentation linked to inflammation and immune editing (84). The presented repertoire also includes cryptic and noncanonical antigens arising from alternative reading frames, aberrant splicing, noncoding regions, defective translation, and related processes that may not be captured by conventional mutation-centered pipelines (55, 85, 86).

Clinical translation nevertheless faces important constraints. Failure to detect a peptide by mass spectrometry does not prove absence because low-abundance ligands may fall below analytical sensitivity; detection does not establish T-cell immunogenicity; and a peptide found in one region may not represent the whole tumor. Conversely, prediction algorithms can generate many false-positive candidates because HLA binding is only one step between a mutation and an effective T-cell target. The most rigorous strategy is therefore orthogonal: sequence the tumor, confirm expression, assess HLA presentation when feasible, test T-cell recognition, and determine clonal and spatial distribution before committing to a personalized therapeutic target (53, 71, 84).

### 6.3 Artificial intelligence and neoantigen prediction

Machine-learning and deep-learning models can improve prediction of peptide-HLA presentation and immunogenicity by integrating sequence features, HLA eluted-ligand data, peptide abundance, and experimentally validated immune responses. NetMHCpan integrates binding and mass-spectrometry ligand data; the TESLA consortium showed that incorporating presentation and recognition features improves ranking of immunogenic candidates; and newer deep neural networks such as BigMHC further improve class I presentation prediction and transfer learning for neoepitope immunogenicity (54, 71, 87, 88).

The major translational risk is overinterpreting computational rank as biological truth. Performance varies across HLA alleles, peptide lengths, tumor types, training datasets, and assay definitions of immunogenicity; data leakage, non-independent benchmarks, and underrepresentation of rare HLA alleles can further inflate apparent accuracy. Because truly immunogenic candidates are rare, severe class imbalance can make area under the receiver-operating-characteristic curve appear favorable despite poor performance among clinically relevant positive candidates. Precision-recall performance, positive predictive value at a clinically usable candidate-selection threshold, and calibration should therefore accompany discrimination metrics. A clinically useful model should ultimately be externally and prospectively validated against direct HLA presentation and functional T-cell assays and should improve therapeutic target selection rather than computational ranking alone (53, 71, 87, 88).

### 6.4 Therapeutic modulation beyond immunotherapy

Chemotherapy and radiation therapy can induce immunogenic cell death, tumor-antigen release, and inflammatory signals that enhance antigen uptake and presentation (42, 43). Targeted therapies may alter HLA expression, antigen-processing machinery, immune-cell infiltration, and the TME, whereas endocrine therapy can alter tumor differentiation and signaling states with potential consequences for immune context (89, 90). Antibody-drug conjugates bridge antigen-directed recognition and cytotoxic drug delivery; their efficacy depends on antigen density, accessibility, internalization, bystander effects, and heterogeneity (44, 91, 92).

### 6.5 Safety and therapeutic constraints of antigen-directed therapy

Therapeutic actionability requires a favorable safety profile as well as target expression and immune recognition. Surface or peptide targets shared with essential normal tissues can produce on-target/off-tumor injury, while engineered T-cell receptors may cross-react with structurally related peptides not predicted during target selection. Serious cardiac toxicity caused by cross-recognition of a titin-derived peptide after affinity-enhanced MAGE-A3 TCR therapy illustrates the importance of rigorous preclinical specificity testing (93). Checkpoint blockade has a different safety profile, with immune-related adverse events resulting from loss of physiological immune tolerance (41).

Cellular therapies and T-cell-engaging bispecific antibodies may produce cytokine-release syndrome and neurotoxicity, in addition to target-dependent normal-tissue injury; antigen density, tumor burden, dose, and TME context influence this therapeutic window (77, 78). Antibody-drug conjugates combine target-dependent toxicity with payload-related and bystander effects (44). Combination regimens may further generate additive or synergistic toxicity. Accordingly, antigen selection for precision therapy should incorporate normal-tissue expression, cross-reactivity, target density, therapeutic index, and the feasibility of managing mechanism-specific adverse events rather than defining actionability from tumor expression alone.

These interconnected processes—from tumor-antigen discovery and biomarker identification to modality-specific therapeutic intervention, safety assessment, and durable disease control—are integrated conceptually in **Figure 2**. **Table 2** then compares the principal therapeutic strategies, their relationship to tumor antigens, biological requirements, and safety constraints.

**Figure 2:**
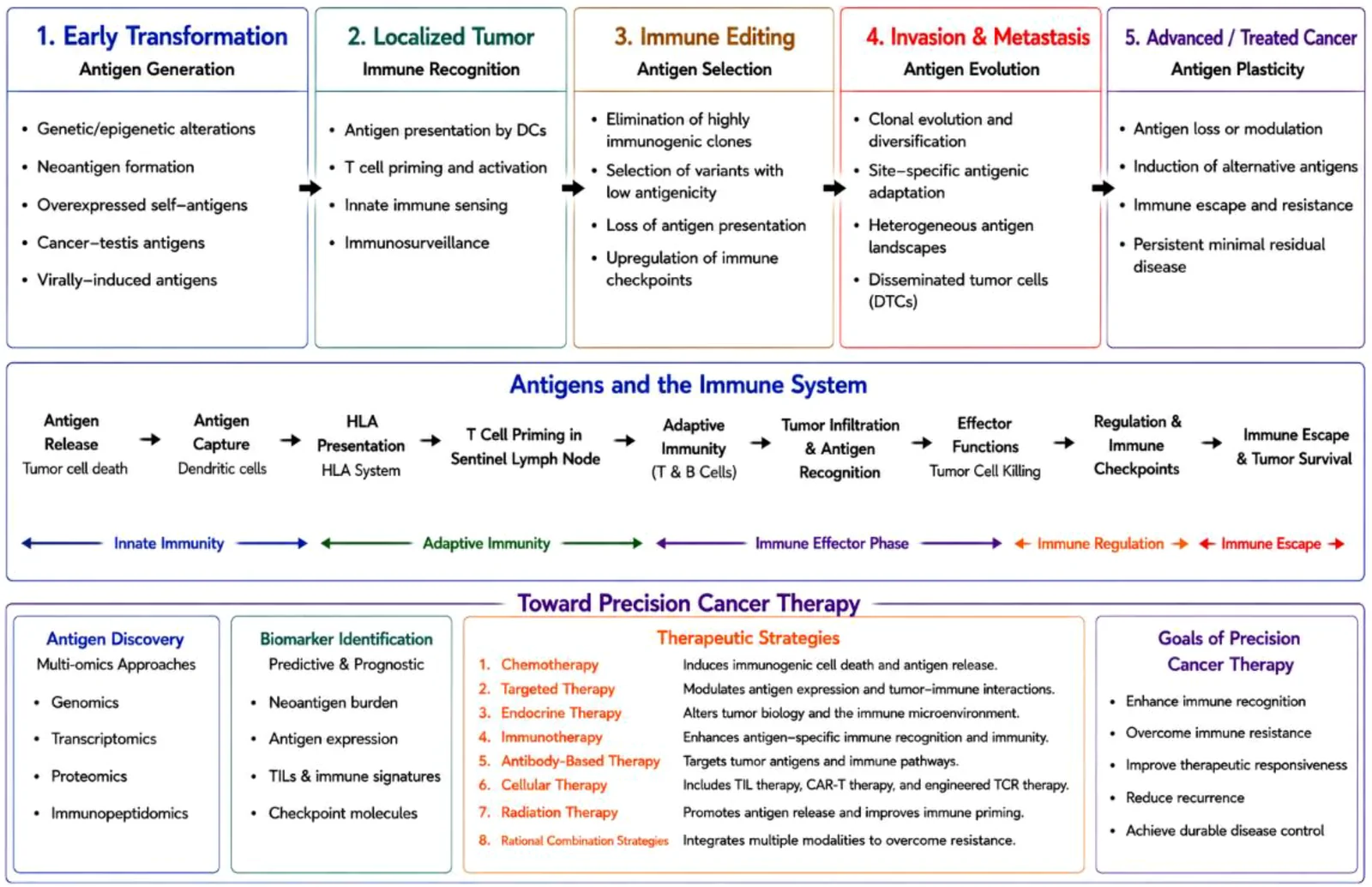
Translating tumor-antigen biology into precision cancer therapy. The figure integrates the biological determinants of tumor-antigen visibility and immune recognition with the downstream processes that shape therapeutic actionability and precision cancer therapy. The upper row depicts five interconnected states of tumor-antigen evolution across cancer progression. 1. Early Transformation is characterized by antigen generation, including genetic/epigenetic alterations, neoantigen formation, overexpressed self-antigens, cancer-testis antigens, and virally induced antigens. This is followed by 2. Localized Tumor, in which immune recognition is mediated through antigen presentation by DCs, T-cell priming and activation, innate immune sensing, and immunosurveillance. The pathway then proceeds to 3. Immune Editing, characterized by antigen selection, including elimination of highly immunogenic clones, selection of variants with low antigenicity, loss of antigen presentation, and upregulation of immune checkpoints. Subsequently, 4. Invasion and Metastasis is associated with antigen evolution, including clonal evolution and diversification, site-specific antigen adaptation, heterogeneous antigen landscapes, and dissemination of tumor cells, including disseminated tumor cells (DTCs). Finally, 5. Advanced/Treated Cancer is characterized by antigen plasticity, including antigen loss or modulation, induction of alternative antigens, immune escape and resistance, and persistence of minimal residual disease. The arrows connecting these five boxes indicate biological progression and selection while recognizing that these states may overlap and need not occur as a strictly linear sequence. The middle row, entitled "Antigens and Immune System," depicts the sequential interaction between tumor-derived antigens and host antitumor immunity. The pathway begins with antigen release following tumor-cell death, followed by antigen capture by dendritic cells and subsequent HLA presentation through the HLA system. Antigen presentation then leads to T-cell priming in the sentinel lymph node, followed by the development of adaptive immunity involving T and B cells. Activated immune cells subsequently undergo tumor infiltration and antigen recognition, leading to effector functions and tumor-cell killing. These responses are further shaped by regulation and immune checkpoints, which can restrain or suppress effective antitumor immunity. Failure of immune recognition, inadequate effector activity, or dominant inhibitory regulation can ultimately result in immune escape and tumor survival. The directional arrows emphasize that these events form an interconnected antigen -immune pathway in which the final biological outcome depends on the integrity and effectiveness of multiple successive steps. Beneath this sequence, overlapping functional immune phases are indicated by bidirectional arrows to emphasize that these processes are not strictly discrete and may operate concurrently. Innate immunity spans the earliest portion of the pathway, extending from antigen release through the early antigen-capture phase. Adaptive immunity begins at HLA presentation and extends through T-cell priming in the sentinel lymph node and the transition toward adaptive T- and B-cell responses. The immune effector phase begins with adaptive immunity and extends through tumor infiltration, antigen recognition, and much of the period of effector function and tumor-cell killing. Immune regulation overlaps the latter portion of the effector phase and extends across regulation and immune checkpoints, reflecting the continuous modulation of antitumor responses by inhibitory and stimulatory mechanisms. Finally, immune escape occupies the terminal portion of the pathway, beginning as regulatory and checkpoint mechanisms predominate and extending through immune escape and tumor survival. The distinct colors of these bidirectional arrows differentiate the major functional phases while emphasizing their biological overlap. The lower translational row links these biological processes to antigen discovery, biomarker identification, therapeutic strategies, and the goals of precision cancer therapy. Antigen discovery incorporates multi-omics approaches including genomics, transcriptomics, proteomics, and immunopeptidomics. Biomarker identification includes predictive and prognostic assessment of neoantigen burden, antigen expression, tumor-infiltrating lymphocytes, immune signatures, and checkpoint molecules. Therapeutic strategies include chemotherapy, which can induce immunogenic cell death and antigen release; targeted therapy, which can modulate antigen expression and tumor -immune interactions; endocrine therapy, which can alter tumor biology and the immune microenvironment; immunotherapy, which can enhance antigen-specific immune recognition and immunity; antibody-based therapy, which can target tumor antigens and immune pathways; cellular therapy, including tumor-infiltrating lymphocyte therapy, CAR-T-cell therapy, and engineered T-cell receptor therapy; radiation therapy, which can promote antigen release and improve immune priming; and rational combination strategies, which integrate multiple modalities to overcome resistance. The ultimate goals of precision cancer therapy are to enhance immune recognition, overcome immune resistance, improve therapeutic responsiveness, reduce recurrence, and achieve durable disease control. Together, the figure emphasizes that successful precision cancer therapy requires integration of tumor-antigen characteristics with HLA presentation, immune competence, spatial tumor-microenvironmental context, functional immune phase, treatment-induced selection, and target safety rather than reliance on antigen detection alone. DCs, dendritic cells; HLA, human leukocyte antigen; TILs, tumor-infiltrating lymphocytes; TCR, T-cell receptor; CAR-T, chimeric antigen receptor T-cell therapy; DTCs, disseminated tumor cells.

**Table 2:** Therapeutic strategies influenced by tumor antigens.

Therapeutic strategy	Relationship with tumor antigens	Principal mechanism	Impact on antigenicity/immune response	Principal safety consideration
Immune-checkpoint blockade	Indirect dependence on tumor-antigen recognition	Releases inhibitory constraints on antigen-specific T cells (41, 68, 74)	Requires retained antigen/HLA presentation, T-cell access, and effector competence	*Immune-related adverse events from loss of physiological tolerance*
Cancer vaccines	Direct antigen targeting	Delivery of shared or personalized antigens to expand tumor-reactive T cells (10, 79–83)	Selection quality depends on verified presentation, immunogenicity, and clonal coverage	*Generally limited systemic toxicity; target selection must minimize normal-tissue cross-reactivity*
Cellular therapy	Direct antigen-specific targeting	TILs, engineered TCRs, or CAR T cells (9, 76, 77)	TCR strategies require HLA presentation; CAR strategies require accessible surface antigen and sufficient density	*On-target/off-tumor toxicity; cytokine-release syndrome and neurotoxicity; TCR cross-reactivity*
Monoclonal antibodies/ADCs	Direct surface-antigen targeting	Antibody effector mechanisms or payload delivery (44, 47, 91, 92)	Depends on surface expression, accessibility, internalization, heterogeneity, and bystander effects	*Normal-tissue target expression; infusion reactions; ADC payload and bystander toxicity*
Chemotherapy	Indirect modulation	Immunogenic cell death and antigen release (42)	Can enhance antigen uptake/presentation but may also select resistant clones	*Conventional cytotoxic toxicity and treatment-related immunosuppression*
Radiation therapy	Indirect modulation	Antigen release, inflammatory signaling, and local immune modulation (43)	May improve priming in some contexts; effects depend on dose, field, timing, and TME	*Normal-tissue radiation injury and treatment-field effects*
Targeted/endocrine therapy	Indirect modulation	Changes oncogenic or hormone-dependent signaling (89, 90)	May alter HLA expression, differentiation, antigen profile, and immune context	*Pathway- or hormone-specific toxicities and drug interactions*
Mechanism-based combinations	Multiple coordinated mechanisms	Correct a demonstrated failure point in presentation, trafficking, effector function, or target coverage (18, 46)	Sequence and timing should follow the dominant biological bottleneck	*Additive or synergistic toxicity requiring mechanism-based sequencing and monitoring*

The table distinguishes direct antigen targeting from therapies that indirectly alter antigen release, presentation, or immune context, identifies the biological requirement that must remain intact for each strategy, and summarizes principal safety constraints that influence clinical actionability.

## 7 Tumor antigens, immunity, and therapeutic response across major human cancers

Cross-cancer comparison is most informative when cancers are not ranked simply as ‘hot’ or ‘cold.’ The clinically relevant variables are the source and clonality of antigens, integrity of HLA presentation, spatial organization of immune cells, dominant suppressive mechanism, and whether an antigen can be targeted directly or only indirectly. **Table 3** therefore compares solid tumors according to their dominant antigenic opportunity and bottleneck rather than listing antigen examples alone. The comparison is intentionally centered on solid tumors because the organizing framework maps antigen-TME co-evolution onto lymphatic, nodal, hematogenous, and distant-organ dissemination; hematologic malignancies follow a different anatomical organization.

**Table 3:** Cross-cancer comparison of tumor antigen biology, immune architecture, and therapeutic bottlenecks.

Cancer type	Representative antigen types	Qualitative antigenic potential	Immune architecture	Therapeutic implications	Dominant bottleneck
Melanoma	UV-associated neoantigens; melanocytic differentiation antigens	Often high; clonal neoantigens can be important	Frequently immune-inflamed but spatially heterogeneous; antigen/HLA escape may emerge	Checkpoint blockade, TIL therapy, vaccines	Antigen/HLA loss, exhaustion, exclusion, and treatment-selected escape (62, 76, 81, 94, 95)
Breast cancer	HER2, MUC1, cancer-testis antigens, neoantigens	Variable by subtype	TNBC more immune-infiltrated; structured immune compartments and stromal-immune ecotypes	Pembrolizumab-based chemoimmunotherapy; HER2-directed antibodies/ADCs	Distinguish T-cell immunogenicity from surface targetability; spatial exclusion/heterogeneity (31, 34, 91, 92, 96)
Colorectal cancer	MSI-H frameshift neoantigens; tumor-associated antigens	High in MSI-H/dMMR; lower effective antigenicity in most MSS tumors	MMRd tumors often contain active immune hubs; MSS tumors frequently excluded/suppressed	PD-1 blockade in MSI-H/dMMR; combinations under study in MSS disease	Antigen quantity alone insufficient; priming, spatial organization, and stromal/myeloid barriers are decisive (32, 33, 35, 97, 98)
Lung cancer	Smoking-associated neoantigens; tumor-associated antigens	Often high in smoking-associated NSCLC	Variable immune infiltration; marked evolutionary and regional heterogeneity	PD-1/PD-L1 blockade and biomarker-guided combinations	Neoantigen loss, HLA/presentation defects, and heterogeneous immunopeptidomes (26, 84, 99)
Prostate cancer	PSA, PSMA, PAP, lineage-associated antigens	Generally low to moderate	Often immune-cold with limited spontaneous T-cell infiltration	Sipuleucel-T and antigen-directed combination approaches	Weak priming and suppressive TME rather than complete absence of lineage antigens (46, 100)
Sarcoma	Fusion-derived, cancer-testis, and selected neoantigens	Highly variable by subtype	TLS/B-cell-rich subsets can be immune active	Selected checkpoint blockade and cellular strategies	Strong subtype dependence; antigen expression and immune architecture must be evaluated together (17, 101)
Virus-associated cancers	Viral antigens such as HPV E6/E7	Potentially high when retained and presented	Foreign antigens increase immune visibility but escape can still occur	Vaccines, checkpoint blockade, adoptive T-cell approaches	HLA/presentation loss and local immune suppression can negate intrinsically foreign antigens (2, 3, 49)

The table compares dominant antigenic opportunities and principal barriers to effective antigen-directed therapy across selected solid tumors. Descriptors in the ‘Qualitative Antigenic Potential’ column represent an integrated qualitative synthesis of antigen source, mutational context, presentation potential, immune visibility, and available biological/clinical evidence; they are not standardized quantitative grades or substitutes for tumor mutational burden.

Melanoma combines high ultraviolet-associated mutational burden with frequent immune infiltration, making it a prototype in which clonal neoantigens, checkpoint regulation, and pre-existing T-cell responses can converge. Durable benefit with checkpoint blockade and the activity of TIL therapy show that antigenic richness can be therapeutically exploited (76, 94, 95). The key limitation is often heterogeneity, antigen/HLA loss, exhausted or excluded T cells, and treatment-driven selection. The randomized phase 2b KEYNOTE-942 experience further suggests that prospectively selected personalized neoantigens can contribute to adjuvant therapeutic strategies, although overall-survival evidence remains immature (81).

Breast cancer illustrates why antigenicity must be separated from targetability. Triple-negative tumors are generally more immune-infiltrated than hormone receptor-positive disease and can respond to pembrolizumab-based chemoimmunotherapy (96). HER2, however, is clinically powerful as an antibody and antibody-drug-conjugate target even when it does not behave as a highly immunogenic neoantigen. Spatial studies further reveal structured immune compartments and stromal-immune ecotypes that can modulate response independently of mutation burden (31, 34). Thus, the dominant question in breast cancer may be whether an antigen is a safe surface address, an HLA-presented T-cell target, or merely a marker of a particular tumor state.

Colorectal cancer provides a sharper contrast within one disease. MSI-H/dMMR tumors accumulate frameshift-derived neoantigens and commonly contain active T-cell responses, explaining their sensitivity to PD-1 blockade (97, 98). Most microsatellite-stable tumors remain resistant despite harboring tumor-associated antigens because lower neoantigen quality, T-cell exclusion, myeloid and stromal programs, and unfavorable spatial organization can become dominant. Single-cell and spatial studies identify immune hubs and cellular neighborhoods that distinguish productive from ineffective immune responses (32, 33, 35). Antigen quantity alone therefore does not explain the MSI-H/MSS therapeutic divide.

Lung cancer demonstrates the interaction between smoking-associated mutational burden and evolutionary immune escape. Many non-small-cell lung cancers generate numerous candidate neoantigens and respond to PD-1/PD-L1 blockade (99). TRACERx analyses nevertheless show that immune pressure can select loss of clonal neoantigens and other escape mechanisms during tumor evolution (26). Immunopeptidomic studies further demonstrate that presented peptides vary among regions and with inflammatory state (84). Thus, high mutational burden can coexist with substantial antigen-presentation and spatial heterogeneity.

Prostate cancer generally has lower mutational burden and limited lymphocytic infiltration, but the randomized double-blind phase III sipuleucel-T trial in 512 patients showed that low baseline immunogenicity does not preclude clinically meaningful antigen-directed immune intervention: median overall survival was 25.8 versus 21.7 months (HR, 0.78; 95% CI, 0.61–0.98), while time to objective disease progression was similar between groups (100). The dominant bottleneck is often insufficient endogenous priming and a suppressive TME rather than complete absence of lineage antigens. Sarcomas are heterogeneous: selected subtypes contain fusion-derived or cancer-testis antigens, and TLS/B-cell-rich subsets show greater checkpoint responsiveness (17, 101). Virus-associated cancers differ again because foreign viral proteins can provide shared non-self targets, although persistence still depends on HLA presentation and immune escape (2, 3, 49).

Pancreatic cancer provides an additional example in which generally low baseline immunogenicity can coexist with vaccine-induced neoantigen-specific T-cell responses, although current personalized-vaccine evidence remains early and highly selected (82). Outside the solid-tumor scope of **Table 3**, CD19-negative relapse after CD19-directed CAR T-cell therapy and BCMA loss after BCMA-directed cellular therapy provide particularly clear independent examples of therapeutic selection for antigen-loss variants (102, 103). These hematologic observations support the broader evolutionary principle without requiring their incorporation into the anatomical solid-tumor comparison.

The comparison therefore supports a general principle: the same therapeutic strategy can fail for different biological reasons in different cancers. In melanoma the problem may be antigen/HLA escape after strong immune selection; in MSS colorectal cancer it may be ineffective priming or exclusion; in prostate cancer it may be weak spontaneous immunity; in HER2-positive breast cancer the actionable feature may be surface antigen density rather than T-cell immunogenicity; and in selected sarcomas local TLS organization may be decisive. Precision therapy should identify the dominant antigen-TME bottleneck rather than assume that a single antigenicity metric applies across cancers.

## 8 Future directions: realizing the potential of tumor antigens in precision cancer therapy while overcoming biological and translational challenges

Modern immunotherapy validates the principle that immune-mediated control of malignancy can be therapeutically exploited, yet many patients do not respond and others develop resistance (45, 46, 104). The central future problem is therefore not simply finding more antigens. It is determining which antigens are truly presented, clonally distributed, immunogenic, spatially accessible, sufficiently safe to target, and stable enough to support durable therapy.

### 8.1 Bottleneck 1: longitudinal and multisite antigen profiling

A single pretreatment biopsy provides only a snapshot. Primary tumors, sentinel and regional lymph nodes, distant metastases, residual disease, and recurrent lesions can differ in clonal composition and immune selection (27, 29, 30, 56, 73). Future studies should prospectively pair baseline and on-treatment tissue with blood-based monitoring and, where clinically justified, biopsy of progressing lesions. Repeated tissue biopsy provides spatial and mechanistic information but carries procedural risk, patient burden, consent considerations, tissue-access limitations, and cost. Blood-based approaches, including ctDNA, circulating tumor cells, circulating immune-cell profiling, and soluble immune markers, offer more scalable longitudinal monitoring, although they cannot fully reproduce spatial information from tissue. The objective is to determine whether antigen loss, HLA loss, immune exclusion, or a suppressive TME emerges before clinical progression rather than reconstructing the mechanism only after treatment has failed.

### 8.2 Bottleneck 2: converting predicted antigens into verified targets

Computational prediction should be treated as triage, not proof. Candidate prioritization should integrate mutation clonality, RNA expression, peptide processing, HLA genotype, direct immunopeptidomic evidence when feasible, and functional T-cell assays (51–53, 71). For clinical development, a practical performance metric is the positive predictive value at the therapeutic selection threshold: the fraction of selected candidates that are demonstrably presented, immunogenic, safe, and retained in the tumor. Emerging immunopeptidomics-guided approaches in lung cancer illustrate how direct presentation data can improve functional neoantigen yield (86).

### 8.3 Bottleneck 3: linking spatial biology to antigen function

Single-cell and spatial datasets should be used to answer explicit causal questions. If an antigen is present but T cells are excluded by stroma or abnormal vasculature, increasing antigen quantity may not help. If T cells are present but the antigen-HLA complex is absent, checkpoint blockade alone may not restore recognition. If antigen-specific T cells and presented antigen coexist but myeloid or metabolic suppression dominates, a different intervention is required. Spatial technologies are therefore most useful when they localize the step at which the antigen-to-effector sequence is interrupted (31–35).

### 8.4 Bottleneck 4: prospective validation of AI-based antigen selection

Artificial intelligence should move from retrospective ranking to prospective clinical validation. Models should be locked before testing, evaluated in independent populations, reported across common and rare HLA alleles, and compared with simpler baselines. Because positive immunogenic candidates are uncommon, prospective studies should report precision-recall performance, positive predictive value at clinically usable selection thresholds, and calibration in addition to conventional discrimination metrics. Where feasible, predicted presentation should be checked against immunopeptidomics and predicted immunogenicity against functional T-cell assays. A useful model should increase the probability that a manufactured vaccine or cellular product targets a biologically relevant antigen; prediction accuracy without demonstrable therapeutic enrichment is insufficient (54, 71, 87, 88).

### 8.5 Bottleneck 5: distinguishing prognostic association from therapeutic actionability

High tumor mutational burden, PD-L1 expression, T-cell density, or a favorable spatial immune pattern may correlate with outcome without identifying the mechanism that limits treatment in an individual patient (45, 70, 105). Biomarkers should therefore be tested for a defined clinical use: prognosis, treatment selection, target confirmation, or pharmacodynamic monitoring. For antigen-directed therapy, the strongest biomarker chain links antigen presence to HLA presentation, immune recognition, target coverage, and clinical response.

### 8.6 Bottleneck 6: designing mechanism-based combination therapy

Combination therapy should correct a demonstrated barrier rather than add agents empirically. An antigen-rich but T-cell-excluded tumor may require improved trafficking or stromal modification; an antigen-presenting tumor with exhausted T cells may benefit from checkpoint modulation; an HLA-deficient tumor may require a strategy that does not depend on conventional peptide-HLA recognition; and a heterogeneous surface-antigen target may require bystander-capable antibody-drug conjugates or multi-antigen targeting. The timing of these interventions should follow the biology of the resistance mechanism rather than a fixed assumption that all barriers are present simultaneously.

These six bottlenecks are not intended as a universal hierarchy. Their relative importance will vary by tumor type, treatment, anatomical compartment, and the dominant biological failure point in an individual patient. A pragmatic translational sequence is to verify antigen expression and presentation, establish target safety, develop feasible longitudinal monitoring, identify the dominant spatial or immune barrier, validate computational prioritization, and then select mechanism-based combination therapy.

Together, these priorities convert future directions from a technology list into a series of testable translational questions: Which antigen is present? Is it clonally expressed? Is it processed and displayed? Which immune cells recognize it? Can they reach the cancer cells? What suppresses their function? Is the target sufficiently selective to be treated safely? Does the target persist during therapy? Can the selected intervention modify the limiting step safely and effectively? Answering these questions longitudinally should make tumor-antigen biology more clinically actionable while preventing overinterpretation of sequence-based antigenicity alone.

## 9 Discussion and conclusions

Tumor antigens are essential determinants of immune recognition, but they do not operate independently of the tissues in which cancers evolve. The evidence reviewed here supports a reciprocal antigen-TME co-evolution model in which cancer-cell antigenic states shape immune and stromal responses, while those host compartments select which antigenic states persist. This reciprocal process occurs across primary tumors, lymphatic and nodal compartments, distant metastatic sites, treatment-altered tissues, residual disease, and recurrence.

Several limitations should be acknowledged. This is a narrative rather than systematic review, and literature selection therefore reflects a single-author synthesis and may be subject to selection bias despite efforts to include supportive, conflicting, and limiting evidence. The available single-cell and spatial literature is unevenly distributed across cancer types and derives largely from selected cohorts studied at specialized academic centers, limiting generalizability of specific spatial architectures and biomarkers across populations, disease stages, and health-care settings. In addition, several elements of the proposed compartment-based antigen-TME framework require prospective longitudinal validation before they can be used routinely for patient selection or treatment design.

The practical implication is that an antigen should be considered clinically actionable only after the relevant biological chain has been examined: expression and clonality, processing and HLA presentation, immune recognition, spatial access, effector competence, normal-tissue safety, and persistence under treatment. Single-cell and spatial technologies, immunopeptidomics, and AI-based prediction can each contribute to this assessment, but none is sufficient alone. Their greatest value will come from integrated, prospective, functionally validated use. Precision cancer immunotherapy can then move from identifying candidate antigens to identifying the specific antigen-TME interaction that can be safely and effectively modified to improve durable disease control.
