# Supplementary material for: Tumor antigens across cancer progression: from immune recognition and selection to precision cancer immunotherapy
Source: Front Immunol. 2026 Oct 12;17:1939919. doi: 10.3389/fimmu.2026.1939919 (PMC13697609; doi:10.3389/fimmu.2026.1939919)
Supplement: Supplementary file 1 [file Table1.docx]

Supplementary Material

**Supplementary Table S1. Cellular Architecture in the Tumor Microenvironment (TME) During Cancer Progression and Dissemination**

| **TME** | **Major Cellular Components** | **Functional Significance** | **Representative References** |
| --- | --- | --- | --- |
| **Primary Tumor TME** | Heterogeneous malignant clones; CD8+ and CD4+ T cells; Tregs; B cells; dendritic cells; macrophages; neutrophils; NK cells; MDSCs; CAFs; endothelial cells; lymphatic endothelial cells; ECM | Tumor-antigen generation, antigen presentation, immune recognition, immune editing, stromal activation, angiogenesis, lymphangiogenesis, and local immune suppression | (8, 13, 29) |
| **Tumor–Lymphatic Interface TME** | Invasive tumor cells near lymphatic vessels; lymphotropic tumor-cell populations; dendritic cells; macrophages; lymphocytes; lymphatic endothelial cells; peritumoral stromal cells; ECM | Tumor-antigen drainage, dendritic-cell migration, immune-cell trafficking, lymphatic invasion, and early movement toward sentinel lymph nodes | (36, 37) |
| **Sentinel Lymph Node TME** | Tumor-derived antigens; isolated tumor cells; micrometastases; dendritic cells; naïve and activated T cells; B cells; macrophages; NK cells; Tregs; lymphatic sinuses; HEVs; nodal stromal cells | First organized regional TME for antigen presentation, lymphocyte activation, immune regulation, tolerance, immune editing, and early nodal metastatic colonization | (21, 22, 36) |
| **Regional Lymph Node TME** | Expanding nodal metastases; antigen-divergent metastatic clones; activated and exhausted T cells; B cells; macrophages; dendritic cells; Tregs; MDSCs; remodeled nodal stroma | Progressive nodal immune selection, metastatic expansion, antigenic diversification, immune suppression, and potential further dissemination | (36-38) |
| **Circulation-Associated TME** | CTCs; tumor-cell clusters; platelets; neutrophils; monocytes; NK cells; circulating lymphocytes; vascular endothelium; soluble cytokines; extracellular vesicles | Tumor-cell survival, immune evasion, platelet shielding, vascular arrest, and distant metastatic seeding | (29, 37, 38) |
| **Premetastatic Niche TME** | Tumor-derived extracellular vesicles and soluble factors; rare disseminated tumor cells; myeloid cells; macrophages; neutrophils; Tregs; activated fibroblasts; endothelial cells; remodeled ECM | Organ conditioning, immune modulation, vascular remodeling, stromal activation, and creation of a permissive environment for metastatic colonization | (13, 29, 39) |
| **Distant Metastatic TME** | Organ-adapted metastatic clones; antigen-divergent and therapy-resistant subclones; CD8+ T cells; exhausted T cells; Tregs; B cells; macrophages; neutrophils; dendritic cells; NK cells; organ-specific stromal cells | Metastatic outgrowth, immune escape, organ-specific adaptation, therapeutic response, and resistance | (25, 29, 39) |
| **Therapy-Associated TME** | Residual tumor cells; resistant clones; antigen-loss variants; dormant cells; exhausted and memory T cells; Tregs; macrophages; dendritic cells; MDSCs; fibrotic stroma; damaged vasculature | Therapy-induced antigen release, immunogenic cell death, immune reactivation or suppression, minimal residual disease, dormancy, recurrence, and resistance | (4, 31, 32) |

**Legend for Supplementary Table S1. Cellular Architecture in the TME During Cancer Progression and Dissemination.** This table summarizes major tumor, immune, stromal, vascular, and lymphatic cellular populations within distinct TMEs that emerge during cancer progression and dissemination, including the primary tumor TME, tumor–lymphatic interface TME, sentinel lymph node TME, regional lymph node TME, circulation-associated TME, premetastatic niche TME, distant metastatic TME, and therapy-associated TME. These cellular components regulate tumor growth, invasion, antigen presentation, immune surveillance, immune escape, metastatic colonization, therapeutic response, dormancy, recurrence, and resistance. Selected representative references are provided for each TME.

**Supplementary Table S2. Tumor Antigens and Molecular Programs in the TME During Cancer Progression and Dissemination**

| **TME** | **Representative Antigenic / Molecular Features** | **Functional Significance** | **Representative References** |
| --- | --- | --- | --- |
| **Primary Tumor TME** | Neoantigens; tumor-associated antigens; differentiation antigens; cancer-testis antigens; viral antigens; HLA class I/II; β2-microglobulin; PD-L1; TMB; MSI; antigen-processing machinery; IFN-γ; IL-10; TGF-β; PD-1/PD-L1; CTLA-4; LAG-3; TIM-3 | Immune recognition, antigen presentation, immune editing, stromal activation, angiogenesis, metabolic adaptation, and local immune suppression | (3, 4, 13) |
| **Tumor–Lymphatic Interface TME** | Tumor antigens entering lymphatic drainage; antigen-bearing extracellular vesicles; lymphotropic tumor-cell profiles; lymphatic remodeling; CCL21/CCR7-associated trafficking; dendritic-cell migration | Antigen drainage, lymphatic dissemination, dendritic-cell trafficking, immune-cell migration, and early immune conditioning | (36, 37) |
| **Sentinel Lymph Node TME** | Tumor-derived antigens; neoantigens; tumor-associated antigens; micrometastatic antigen sources; HLA class I/II; dendritic-cell activation; CD8; CD4; FoxP3; B-cell markers; CXCL13; CCL19; CCL21; PD-1/PD-L1; CTLA-4 | Antigen presentation, T-cell priming, B-cell activation, immune tolerance, immune editing, and early nodal metastasis | (21, 22, 36) |
| **Regional Lymph Node TME** | Antigen-divergent nodal metastatic clones; retained or lost tumor antigens; therapy-selected antigenic profiles; PD-L1; HLA class I/II; β2-microglobulin; CD8; FoxP3; macrophage markers; B-cell markers; IL-10; TGF-β; VEGF | Nodal immune editing, metastatic expansion, immune exhaustion, antigen-loss selection, immune suppression, and lymphovascular remodeling | (4, 36, 37) |
| **Circulation-Associated TME** | CTC antigen profiles; antigen-low or immune-resistant circulating clones; tumor-derived extracellular vesicles; ctDNA; PD-L1-positive CTCs; platelet-coated CTCs; inflammatory mediators; epithelial–mesenchymal plasticity | CTC survival, immune evasion, platelet shielding, vascular arrest, and distant metastatic seeding | (25, 37, 38) |
| **Premetastatic Niche TME** | Tumor-derived extracellular-vesicle antigens; disseminated tumor-cell signals; organ-conditioning factors; VEGF; TGF-β; IL-6; CXCL12; CCL2; myeloid-cell recruitment; stromal remodeling; vascular permeability | Organ-specific niche conditioning, immune suppression, vascular remodeling, stromal activation, and metastatic permissiveness | (13, 29, 39) |
| **Distant Metastatic TME** | Organ-adapted antigens; neoantigen-loss variants; antigen-low clones; therapy-resistant profiles; PD-L1; HLA class I/II; β2-microglobulin; TMB; MSI; immune-gene signatures; CXCL9; CXCL10; CXCL13; PD-1/PD-L1; CTLA-4; LAG-3; TIM-3; TIGIT; VISTA | Metastatic colonization, immune escape, organ-specific adaptation, angiogenesis, metabolic adaptation, and therapy resistance | McGranahan and Swanton, 2017; Havel 2019; Hanahan, 2022 |
| **Therapy-Associated TME** | Therapy-induced antigen release; antigen-loss variants; resistant clones; dormant antigen-low cells; newly exposed neoantigens; PD-L1; HLA class I/II; antigen-processing machinery; ctDNA; CTCs; IFN signaling; checkpoint upregulation; T-cell exhaustion; myeloid suppression | Antigen spreading, immunogenic cell death, therapy-induced selection, minimal residual disease, dormancy, recurrence, and resistance | (4, 31, 32) |

**Legend for Supplementary Table S2. Tumor Antigens and Molecular Programs in the TME During Cancer Progression and Dissemination.** This table summarizes representative tumor antigens, antigenic features, biomarkers, cytokines, chemokines, immune checkpoints, suppressive pathways, and major biological programs within distinct TMEs that emerge during cancer progression and dissemination, including the primary tumor TME, tumor–lymphatic interface TME, sentinel lymph node TME, regional lymph node TME, circulation-associated TME, premetastatic niche TME, distant metastatic TME, and therapy-associated TME. These molecular and antigenic programs reflect bidirectional co-evolution between tumor cells and the TME, driving antigen presentation, immune editing, clonal selection, immune escape, metastatic fitness, therapeutic responsiveness, dormancy, recurrence, and resistance. Selected representative references are provided for each TME.
